# Use of a Modified Transhepatic Rendezvous Technique for Stenting of Malignant Biliary Obstruction in 2 Cats

**DOI:** 10.1111/jvim.70178

**Published:** 2025-07-04

**Authors:** Robin Doherty, Allyson C. Berent, Chick Weisse

**Affiliations:** ^1^ Department of Interventional Radiology and Endoscopy/Internal Medicine Schwarzman Animal Medical Center New York New York USA; ^2^ Department of Interventional Radiology and Endoscopy Schwarzman Animal Medical Center New York New York USA

**Keywords:** bile duct rupture/obstruction, biliary rendezvous, biliary stent, gastroenterology, hepatology, malignant biliary obstruction

## Abstract

We describe the successful treatment of biliary duct obstruction (BDO) secondary to biliary carcinoma involving the bile duct, cystic duct, and intrahepatic ducts in two adult domestic shorthair cats using a novel technique. Both cats presented with marked hyperbilirubinemia, increased liver enzyme activities, and ultrasonographic evidence of BDO secondary to neoplasia. One cat previously had undergone cholecystoduodenostomy, with recurrence of BDO. Both cats were treated using a transhepatic biliary rendezvous procedure, in which a hybrid interventional radiological approach was used to place a self‐expanding metallic stent into the left intrahepatic duct, into the common bile or cystic duct, and into the duodenum, relieving the obstruction. In both cases, BDO resolved at time of last follow‐up. This technique offers an alternative to traditional surgical or interventional options for biliary decompression, particularly in cases of diffuse malignant biliary obstruction where access to the common bile duct (CBD) is challenging.

**Abbreviations**
ALP: alkaline phosphatase
ALT: alanine transaminase
BDO: biliary duct obstruction
CBD: common bile duct
CCE: cholecystenterostomy
EHBDO: extraheptic biliary duct obstruction
ERC: endoscopic retrograde cholangiography
ERC‐BS: endoscopic retrograde cholangiography with biliary stenting
ERCP: endoscopic retrograde cholangiopancreatography
FTUBE: via feeding tube
MDP: major duodenal papilla
PTBD: percutaneous transhepatic biliary drainage
PTE‐RV: percutaneous transhepatic endoscopic rendezvous
SEMS: self‐expanding metallic stent

## 1 Introduction

Extrahepatic biliary duct obstruction (EHBDO) is associated with a variety of underlying conditions and leads to serious hepatobiliary injury within weeks [1]. A retrospective study of 41 canine and 4 feline cases of EHBDO found that the most common cause was pancreatitis (42%), followed by extraluminal and intraluminal neoplasia (24.3%), cholangitis (17.8%), and cholelithiasis (8.8%). When evaluating the feline cases alone, neoplasia was the most common cause (75%) [2].

Traditional treatment for EHBDO involves laparotomy with biliary diversion or surgical biliary stent placement across the major duodenal papilla (MDP) via duodenotomy. The peri‐operative mortality rates of biliary diversion techniques are reported to be 28%–67% [2, 3, 4, 5, 6] and 41%–57% [5, 7, 8, 9] in dogs and cats, respectively. Open surgical placement of biliary stents has been associated with a peri‐operative mortality rate of 30.7% in dogs [10] and 21.7%–28% in cats [11, 12]. In the last decade, the use of interventional radiology and endoscopy procedures in veterinary patients has been shown to decrease peri‐operative morbidity and mortality [13, 14, 15, 16].

Endoscopic retrograde cholangiopancreatography (ERCP) utilizes endoscopy and fluoroscopy to image the common bile duct (CBD) and pancreatic ducts [13]. This technique has been reported in 44 dogs and cats [13, 14, 15, 16, 17], with success rates of 40%–67%. It has been combined with decompressive biliary stenting (ERC‐BS) or sphincterotomy in one study of 7 research and 2 clinical dogs [16]. More recently, a report of ERCP in 14 clinical dogs and 3 cats, showed successful decompression in 14/17 (82%) dogs and 1/3 (33%) cats [18]. The primary limiting factor is the requirement for cannulation of the CBD, which is especially challenging in patients with obstructive neoplasia at the level at the MDP, effacing the orifice.

An alternative to ERC‐BS is a rendezvous technique in which access to the biliary system is gained through puncture of the liver into an intrahepatic biliary duct, with a guidewire advanced down the CBD and into the duodenum. Once the guidewire is in the duodenum, it can be retrieved and pulled out through the mouth using a gastroduodenoscope and a grasping instrument. If done percutaneously, which is most common in humans, it is called a percutaneous transhepatic rendezvous technique (PTE‐RV) [19]. A single report describes PTE‐RV to treat EHBDO secondary to infectious cholecystitis and mineralized debris in a dog [20]. The dog made a full recovery with stent placement and culture‐based antibiotic treatment.

The following cases describe the first reported use of a modified transhepatic rendezvous technique (modified PTE‐RV) using ultrasonography, fluoroscopy and surgical assistance for biliary stenting in 2 cats with infiltrative and diffuse malignant intrahepatic and extrahepatic biliary duct obstruction (BDO).

## 2 Case Descriptions

### 2.1 Case 1

A 13‐year‐old 4.65 kg male castrated domestic shorthair cat was presented for further evaluation of biliary obstruction secondary to a previously diagnosed biliary carcinoma.

The patient had undergone a computed tomography (CT) scan and exploratory laparotomy, which identified a non‐resectable mass involving the entire CBD and extending to the liver hilus, with subsequent diffuse intrahepatic biliary duct dilatation. Biopsy of the mass was consistent with carcinoma. There also was concern for a soft tissue structure in the right caudal lung lobe and sternal lymphadenopathy on imaging. Incidental findings included left coxofemoral subluxation and a chronic pubic fracture associated with previous trauma. Previous treatment included placement of an esophagostomy tube; a single dose of carboplatin at 180 mg/m^2^ IV; hospitalization for IV fluid therapy, ampicillin/sulbactam, vitamin B complex, vitamin K1, maropitant, cyproheptadine, pantoprazole, and gabapentin; as well as prior administration of robenacoxib, mirtazapine, gabapentin, and a supplement containing silymarin, N‐acetyl cysteine, zinc, and vitamin E.

At presentation 8 days after exploratory laparotomy, test results indicated markedly increased liver enzyme activities and hyperbilirubinemia (Table S1) as well as neutrophilia with a left shift, increased symmetric dimethylarginine (SDMA), hypokalemia, and hypoalbuminemia. Abdominal ultrasonography identified a hyperechoic liver with generalized intrahepatic duct distension without clinically relevant gallbladder dilatation, with a hypoechoic nodule (approximately 0.6 cm) at the level of the distal CBD. Bilateral chronic kidney disease and a small volume of peritoneal effusion also were noted.

**FIGURE 1:**
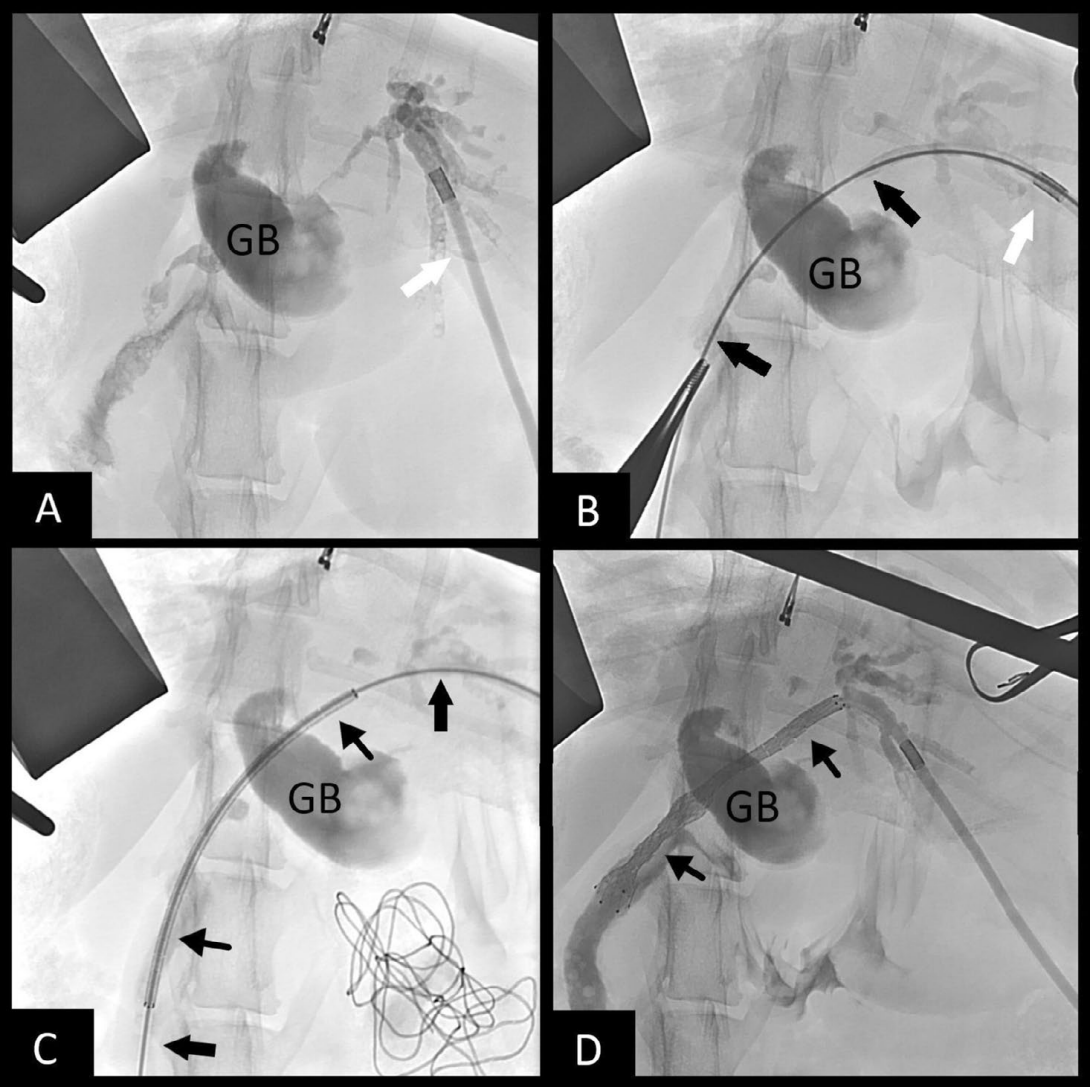
Fluoroscopic images of transhepatic biliary rendezvous technique performed in Case 1. (A)—Fluoroscopic study showing the catheter (white arrow) within the intrahepatic duct, with a successful cholangiogram performed. The gallbladder is denoted as GB. (B)—Passage of the hydrophilic guidewire (thick black arrows) through the CBD and into the duodenum. (C)—Passage of the biliary stent delivery system (thin black arrows) over the wire. (D)—Cholangiogram showing patency of the deployed biliary stent (thinner black arrows) along with final placement of the pigtail catheter within the intrahepatic duct.

Biliary stenting was recommended and carried out. The patient was pretreated with vitamin K1 (0.5 mg/kg SC q 12 h) and deamino‐D‐arginine vasopressin (DDAVP; 1.0 u/kg SC 15 min before surgery) to minimize bleeding risk. Exploratory laparotomy confirmed biliary obstruction secondary to diffuse tumor burden. Gallbladder puncture using an 18‐Ga IV catheter was performed in an attempt to gain biliary access because the entire CBD was filled with tumor, but was unsuccessful because of occlusion of the cystic duct by tumor. Biliary access then was obtained using intra‐operative ultrasound‐guided puncture of a dilated intrahepatic bile duct of the left lateral lobe using a 22‐Ga IV catheter. An antegrade cholangiogram confirmed obstruction from the intrahepatic ducts at the level of the hilus and throughout the entire CBD (Figure 1A). A 0.018″ hydrophilic guidewire (Weasel Wire, angled 0.018″ by 150 cm, Infiniti Medical, Palo Alto, Cali) was passed through the catheter and into the intrahepatic biliary system. The 22‐Ga catheter was exchanged for a 4 French micropuncture system (Micropuncture Access Set, 4Fr by 10 cm, Cook Medical, Bloomington Ill) and the guidewire was exchanged for a 0.035″ stiffened angle‐tipped hydrophilic guidewire (Weasel Wire, stiff angled 0.035″ by 150 cm, Infiniti Medical, Palo Alto, Cali). Under fluoroscopic guidance the guidewire was navigated through the tumor and CBD (Figure 1B) and into the duodenum. A small puncture was made into the descending duodenum to gain through‐and‐through guidewire access. A 6 mm × 60 mm non‐covered self‐expanding metallic stent (SEMS) then was advanced in a retrograde manner over the guidewire from the duodenum up the CBD and into the left intrahepatic biliary duct (Figure 1C). The stent was deployed across the CBD and into the duodenum (Figure 1D) allowing for decompression of the left side of the liver. A locking loop nephrostomy catheter (Locking Loop Catheter with Stiffening Cannula, 6.5F by 20 cm, Norfolk Vet Products, Skokie Ill) was fed over the guidewire from the liver side and coiled into the left biliary duct for potential future intraparenchymal chemotherapy. A dacron cuff was glued into place on the liver capsule to prevent biliary leakage, and the distal end of the catheter was passed through the body wall using blunt dissection and connected to a SC access port (Medium titanium vascular access port, Norfolk Vet Products, Skokie, Ill) for future access. Total procedure time was 97 min. Bile collected during biliary puncture was submitted for aerobic and anaerobic culture, and all cultures were negative.

The cat recovered well and was hospitalized for the next 3 days on IV fluid therapy, ampicillin/sulbactam (30 mg/kg IV q8h), enrofloxacin (5 mg/kg IV q24h), Vitamin K1 (0.5 mg/kg SC q12h), ondansetron (0.5 mg/kg IV q12h), pantoprazole (1 mg/kg IV q12h), ursodiol (13.5 mg/kg via feeding tube [FTUBE] q24h), methadone (0.2 mg/kg IV q6h), vitamin B complex, and metoclopramide (2 mg/kg/day IV).

Hyperbilirubinemia improved by 1 day post‐operatively, with initial progression of increased hepatocellular enzyme activities, followed by gradual decreases on subsequent follow‐up (Table S1).

**FIGURE 2:**
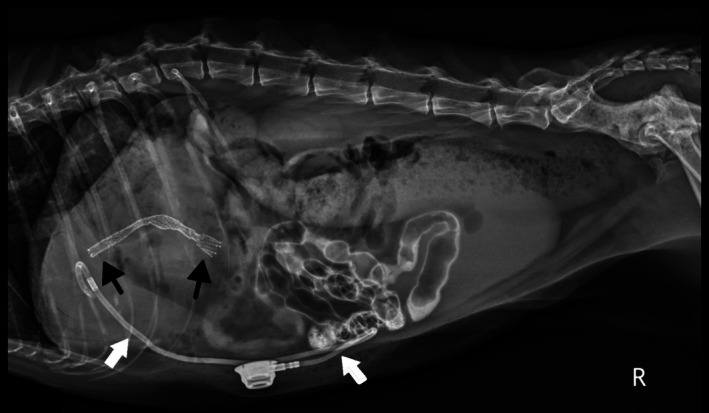
Post‐operative right lateral abdominal radiograph for Case 1. The biliary drainage tube (white arrows) and stent (black arrows) are appropriately placed. There is persistent contrast within the gastrointestinal tract showing patency of the biliary system after the cholangiogram.

**FIGURE 3:**
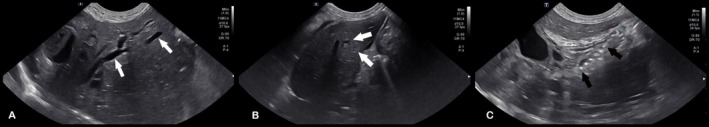
Post‐operative ultrasound images for Case 1. (A)—Right liver with persistently dilated intrahepatic ducts (white arrows). (B)—Left liver with resolution of intrahepatic duct dilation (white arrows). (C)—Stent in place across the CBD (black arrows).

Post‐operative radiographs confirmed appropriate placement of the stent and the biliary drainage catheter (Figure 2). Abdominal ultrasonography showed asymmetric biliary tract decompression, with persistent right‐sided intrahepatic biliary tract dilatation (Figure 3A) and resolved left intrahepatic biliary tract dilatation (Figure 3B). The stent was visualized extending from the duodenal lumen through the CBD and into the left lateral liver lobe within the biliary tract (Figure 3C). A large volume of echogenic peritoneal effusion also was observed with hyperechoic mesenteric fat. Fluid analysis was consistent with mild mixed inflammation; no bacteria were seen; aerobic and anaerobic cultures were negative.

The cat was discharged 3 days post‐operatively and transitioned to enteral medications, including amoxicillin/clavulanic acid (13.5 mg/kg PO q12h), enrofloxacin (4.9 mg/kg PO q24h), ondansetron (0.4 mg/kg PO q12h), ursodiol (13.5 mg/kg PO q24h), and cyproheptadine (0.4 mg/kg PO q12h).

There was initial improvement in clinical signs, alongside improvements in laboratory test results. However, the cat was evaluated for anorexia and regurgitation 15 days post‐operatively. Liver enzyme activities and serum bilirubin concentration had continued to improve (Table S1). Abdominal ultrasonography identified an appropriately placed biliary stent with persistent right intrahepatic duct dilatation, resolution of left hepatic duct dilatation, and mild peritoneal effusion. Gastric distension and the presence of hypochloremic metabolic alkalosis were concerning for ileus without evidence of mechanical obstruction. The patient was hospitalized on IV fluids, potassium supplementation, ampicillin/sulbactam (30 mg/kg IV q8h), enrofloxacin (5 mg/kg IV q24h), doxycycline (5 mg/kg IV q12h), maropitant (1 mg/kg SC q24h), buprenorphine (0.015 mg/kg IV q8h), gabapentin (6 mg/kg FTUBE q12h), pantoprazole (1 mg/kg IV q12h), ondansetron (0.5 mg/kg FTUBE q12h), metoclopramide (2 mg/kg/day IV constant rate infusion [CRI]), cisapride (0.6 mg/kg FTUBE q12h), and thiamine (12.5 mg/kg IM q12h). Regurgitation persisted, and repeat abdominal ultrasonography indicated progressive gastric distension and peritoneal effusion with suspicion of functional ileus or the biliary tumor causing mechanical outflow tract obstruction to the proximal duodenum. The biliary stent was not seen to be obstructing the duodenal lumen. The cat became recumbent, developed refractory hypotension, and was euthanized 19 days post‐operatively. A necropsy was not performed.

### 2.2 Case 2

An 8‐year‐old 3.95 kg male castrated domestic shorthair cat was presented for further evaluation of BDO secondary to a previously diagnosed bile duct adenocarcinoma.

The cat previously had undergone abdominal ultrasonography and exploratory laparotomy, which identified biliary obstruction with marked distension of the gallbladder, hepatic and cystic ducts, and a mass within the CBD causing complete luminal obstruction. The cat was also noted to have pancreatitis and peritonitis. A routine cholecystoduodenostomy was performed. Histopathology of the mass was consistent with bile duct adenocarcinoma. Previous treatment included IV fluid therapy, ampicillin, enrofloxacin, ondansetron, maropitant, famotidine, Denamarin, and vitamins B12 and K1.

Although the patient initially improved after cholecystoduodenostomy with resolution of hyperbilirubinemia, recurrence of the biliary obstruction occurred 16 days later. The cat was presented to the hospital with persistent severe hyperbilirubinemia, increased liver enzyme activities, as well as anemia, reticulocytosis, monocytosis, and an increased plasma fibrinogen concentration.

**FIGURE 4:**
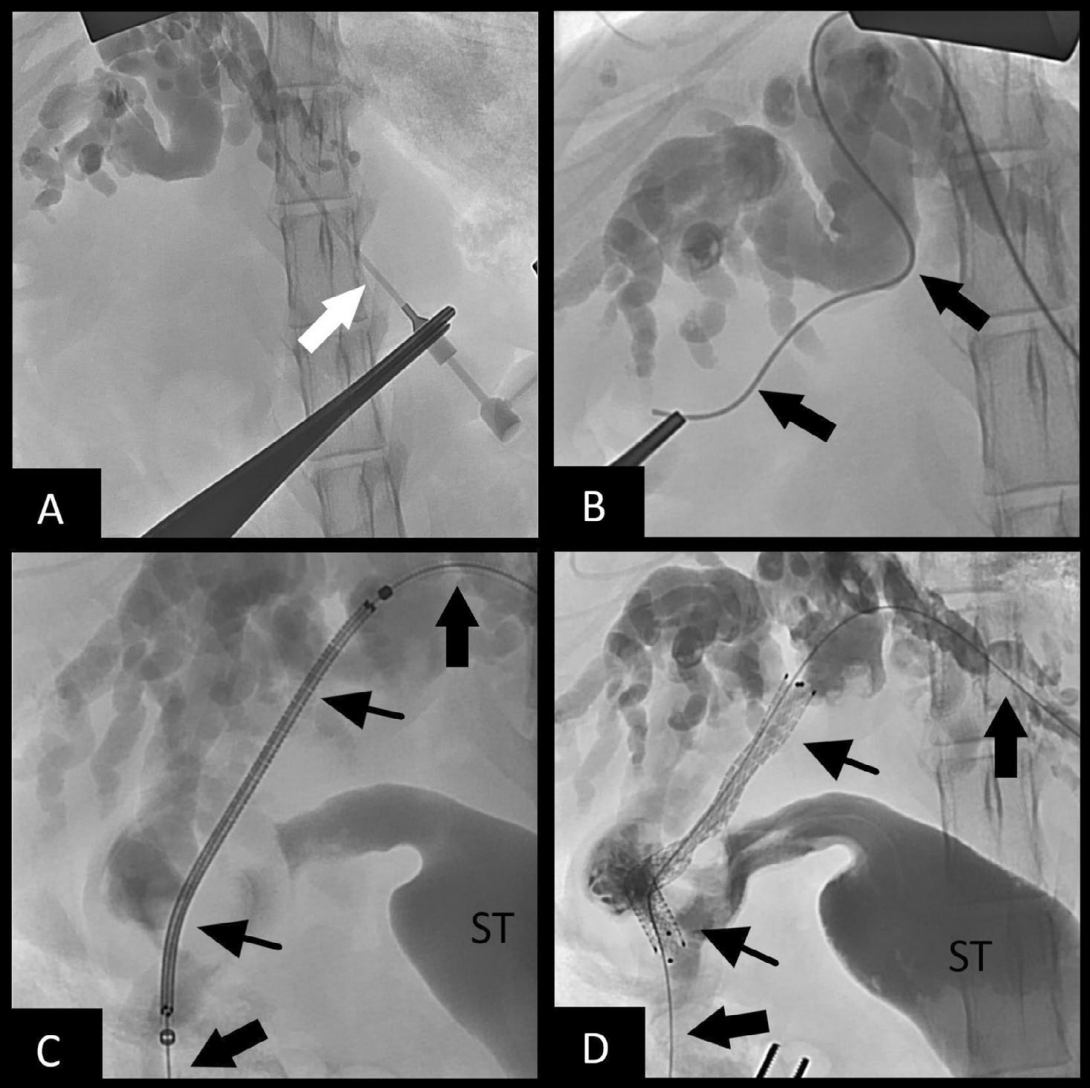
Fluoroscopic images of transhepatic biliary rendezvous technique performed in Case 2. (A)—Fluoroscopic study showing the catheter (white arrow) within the intrahepatic duct, with a successful cholangiogram performed. (B)—Passage of the hydrophilic guidewire (thick black arrows) through the CBD and into the duodenum. (C)—Passage of the biliary stent delivery system (thin black arrows) over the wire. Contrast filling of the stomach (ST) is noted after filling the stent delivery system with contract to document the duodenal lumen location on fluoroscopic imaging for distant stent placement. (D)—Cholangiogram showing patency of the deployed biliary stent (thin black arrows).

Surgical intervention to pursue biliary stenting was considered. Vitamin K1 (0.6 mg/kg SC) and DDAVP (1.1 mg/kg SC) were given preoperatively. On abdominal exploratory, the tumor was noted to be obstructing the cystic duct, leading to failure of the cholecystoduodenostomy. A rendezvous procedure utilizing a hybrid approach was performed as described above, with some modifications. Biliary access was accomplished through a dilated intrahepatic bile duct of the left lateral lobe. An antegrade cholangiogram confirmed obstruction (Figure 4A). The guidewire and micropuncture system were utilized, as described in case 1, but because of the choleduodenostomy, the wire was passed through the cystic duct, into the gallbladder, and out into the duodenum (Figure 4B). Additionally, instead of retrograde stent placement, a non‐covered SEMS biliary stent (4 mm × 60 mm) was placed in an antegrade manner from the left intrahepatic biliary duct access point, through the cystic duct, and exiting the cholecystoduodenostomy site within the gallbladder (Figure 4C), which was communicating with the duodenum. A post‐stenting cholangiogram confirmed biliary patency (Figure 4D). A locking loop catheter again was advanced over the guidewire and placed into the left biliary duct, and it was left in place connected to a SC access port for potential intraparenchymal chemotherapy. Total procedure time was 111 min. The bile culture was positive for *Enterococcus faecium* .

Recovery was uneventful and the cat was treated with IV fluid therapy with supplemental potassium chloride, metoclopramide (2 mg/kg/day IV CRI), maropitant (1 mg/kg IV q24h), ondansetron (0.9 mg/kg IV q12h), ampicillin/sulbactam (30 mg/kg IV q8h), enrofloxacin (5 mg/kg IV q24h), and methadone (0.2 mg/kg IV q6h).

**FIGURE 5:**
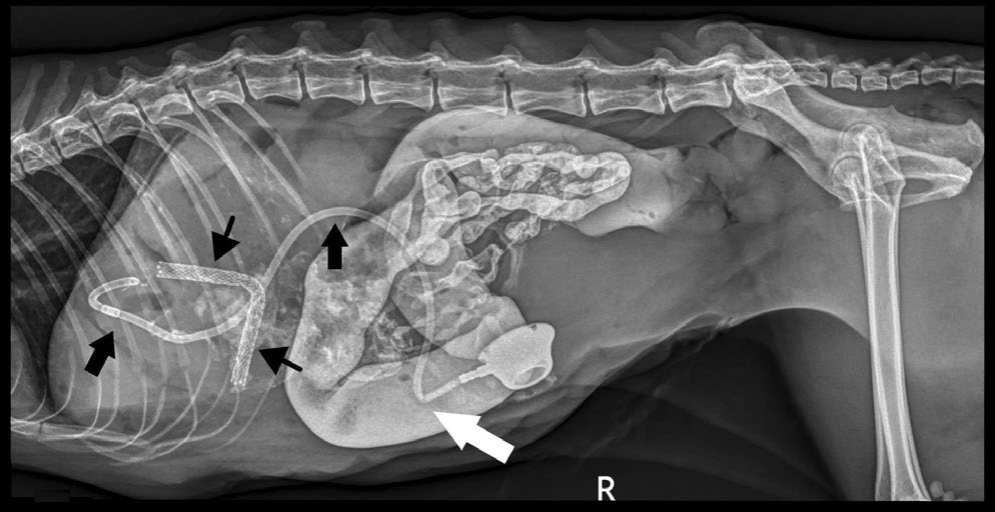
Post‐operative right lateral abdominal radiograph for Case 2. The biliary drainage catheter (thick black arrows) and stent (thin black arrows) are appropriately placed. There is a kink (white arrow) noted in the biliary drainage catheter. There is persistent contrast within the gastrointestinal tract.

**FIGURE 6:**
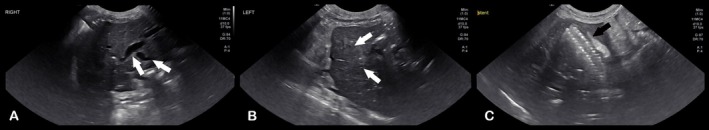
Ultrasound images of the liver and CBD following stent placement. (A)—Right liver with persistently dilated intrahepatic ducts (white arrows). (B)—Left liver with resolution of intrahepatic duct dilation (white arrows). (C)—Stent in place across the cystic duct (black arrow).

Improvement of hyperbilirubinemia and increased liver enzyme activities was noted post‐operatively (Table S1). Post‐operative radiographs showed an appropriately‐placed stent and biliary drainage tube (see Figure 5). A partial kink of the tubing was seen as it entered the body wall, but it was of no clinical consequence. Ultrasonography showed asymmetrical decompression of the intrahepatic biliary duct tracts, with ongoing right biliary obstruction and left‐sided decompression (Figure 6). Echogenic peritoneal effusion and hyperechogenicity of the cranial mesenteric fat also were noted.

The cat was discharged 1 day post‐operatively for continued hospitalization at the referring veterinarian and transitioned to enteral doxycycline based on culture results. Laboratory testing performed 3 days post‐operatively showed improvement in liver enzyme activities and serum bilirubin concentration (Table S1). Three weeks postoperatively the cat was reported to have normal serum bilirubin concentration and liver enzyme activities. A repeat CT scan before scheduled radiation planning showed pulmonary metastasis, and PO chemotherapy was initiated. On day 63 post‐operatively, the owner reported a mild increase in serum total bilirubin concentration above the reference interval, but it was < 2.0 mg/dL and the cat was doing well without evidence of left‐sided biliary dilatation. After this update the patient was lost to follow‐up.

## 3 Discussion

In these cases, a modified version of PTE‐RV was used to treat diffuse malignant biliary obstruction in two cats in which surgical options were limited. To our knowledge, these cases represent the first report of a biliary rendezvous procedure performed in cats.

The outcomes of these cases support that this technique can be used in cats to decompress the biliary system if the intrahepatic biliary ducts are visible on ultrasonography. This procedure offers an alternative to more traditional surgical interventions, such as cholecystenterostomy (CCE) or traditional surgically assisted biliary stenting via enterotomy, especially in cases where malignant obstructions may be occluding the entire biliary tract. This technique was faster technically and less invasive than traditional CCE. In one case, a CCE had been performed and failed, whereas in the other case, the cystic duct was obstructed, making CCE not feasible. A traditional endoscopic retrograde cholangiopancreatography (ERCP) for retrograde stenting was not possible because of tumor location and effacement of the MDP, and because of the small size of the patients.

Limitations of our study include that only partial liver decompression was achieved, only 2 cases were included, and there was limited follow‐up, with disease progressing rapidly. Despite only partial decompression, resolution of the biochemical changes of cholestasis was achieved post‐operatively.

In these 2 cases, one of the left lateral intrahepatic bile ducts was chosen for decompression because the left liver comprised a larger portion of overall hepatic mass, as well as for ease of access during surgery [21]. In humans, partial decompression of the left liver lobe alone, comprising an average of 31% of the human liver [22], resulted in improvement in liver function to near normal after 6 weeks [23].

Another limitation to this technique was the use of an open abdominal approach. An open approach was used to shorten procedure times in these two severely debilitated patients and to avoid risking loss of access to the intrahepatic ducts. The feline liver is much more mobile than a human or canine liver, increasing the risk of biliary leakage post‐puncture. An additional limitation is the fact that only two cats are presented here, both of which had severe intrahepatic and extrahepatic biliary obstruction associated with diffuse neoplasia. The prognosis for malignant biliary obstruction in cats is especially grave [8], with a median survival time of 14 days reported, and most affected cats do not survive to discharge. Despite the severity of the disease in these cats, both survived to discharge. Cat 1 developed severe ileus and regurgitation 15 days post‐operatively. The cause was unclear, and the owner declined necropsy. One consideration was the possibility of an upper duodenal obstruction from gastric contents or hair entrapped by the end of the stent in the lumen of the duodenum, but this possibility was not documented on ultrasonography immediately before euthanasia. Because the biliary stent in cat 2 extended into the gallbladder and not the duodenum (because of previous CCE), this factor may have protected the duodenum from the stent. Cat 2 was still alive at the time of last follow‐up (63 days), but pulmonary metastasis was reported.

## 4 Conclusions

In conclusion, the modified PTE‐RV procedure was technically successful in 2 cats with malignant EHBDO. This technique offers an alternative to traditional surgical treatment, especially when ERC‐BS is not possible. Additional studies are needed to evaluate long‐term outcomes and to assess utility for benign causes of biliary obstruction.

## Disclosure

Authors declare no off‐label use of antimicrobials.

## Ethics Statement

Authors declare no institutional animal care and use committee or other approval was needed. Use of non‐experimental (client‐owned) animals followed best practice standards for each individual cat. Authors declare human ethics approval was not needed.

## Conflicts of Interest

The authors declare no conflicts of interest.

## Supporting information


**Table S1.** Serial blood testing results for both cases, with D0 representing the day the rendezvous procedure was performed. Reference ranges from IDEXX Laboratories Inc.
